# Minimally Invasive Eyelid Lift: A Skin Preservation Upper Blepharoplasty

**DOI:** 10.1093/asjof/ojaf107

**Published:** 2025-08-25

**Authors:** Alice Siew Ching Goh, Geng-Yi Yong

## Abstract

**Background:**

Traditional upper blepharoplasty often involves skin excision, which can lead to complications such as hollowing, visible scarring, and unnatural result. A tissue-preservation approach may offer quicker recovery and improved cosmetic outcomes.

**Objectives:**

In this preliminary report, the authors introduce the Minimally Invasive Eyelid Lift (MINEL), a novel tissue-preserving upper blepharoplasty technique that tucks excess skin inward, avoiding skin excision, and evaluates its effectiveness in achieving natural and lasting results.

**Methods:**

We included patients with dermatochalasis and underwent MINEL procedure. The technique involved creating small incisions, tucking the excess skin inwards, and securing it with nonabsorbable sutures.

**Results:**

Thirty-seven patients undergoing the MINEL procedure all reported positive functional and aesthetic outcomes, with 70% very satisfied. The technique effectively reduced redundant skin, preserved natural eyelid creases, and resulted in minimal scarring. The average procedure duration was 33.9 min, with rapid recovery and return to activities.

**Conclusions:**

MINEL offers a promising tissue-preserving alternative to traditional upper blepharoplasty, providing natural aesthetic outcomes with minimal scarring and faster recovery. Further studies are needed to evaluate long-term results and compare its efficacy, including with traditional techniques, to validate its role in cosmetic eyelid surgery.

**Level of Evidence: 4 (Therapeutic):**

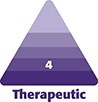

Aging in the periorbital region is often one of the earliest signs of facial aging, with hooded upper eyelids presenting a common aesthetic concern for both men and women. Current trends in upper blepharoplasty emphasize volume preservation, prompting surgeons to prioritize the maintenance of the orbicularis oculi muscle and orbital fat. Traditionally, upper blepharoplasty has involved the excision of skin, muscle, and fat.^1^ Although this approach aims to rejuvenate the eyelids, it can sometimes yield unnatural results, marked by visible scarring.

Because cosmetic surgery increasingly prioritizes tissue preservation, aggressive excision techniques are becoming less common. Surgeons now favor methods that focus on preserving native tissue, particularly in fat compartments. These preservation techniques are often combined with augmentation strategies to enhance a youthful appearance.^2-4^ Complications from upper eyelid blepharoplasty are generally minimal, especially when volume-preserving techniques are used to reduce the risk of upper lid hollowing.^2,4^ Currently, the preferred approach to upper eyelid blepharoplasty involves preserving, repositioning, or modestly filling the upper lid fat.^2,5^ When fat reduction is necessary, a conservative approach is recommended.^6,7^ Additionally, preserving the orbicularis oculi is crucial for maintaining eyelid fullness, a hallmark of a youthful look.^8^

In response to these evolving trends, we introduce the Minimally Invasive Eyelid Lift (MINEL), a novel procedure designed to address upper eyelid redundancy without the need for skin excision. Instead, MINEL repositions and tucks excess skin inward, enhancing or creating a natural eyelid crease while effectively tightening the lid to restore a youthful contour. This technique not only preserves the patient's native tissue but also counters age-related involutional changes, offering subtle and natural enhancements with minimal downtime and scarring. This paper details the MINEL technique and presents preliminary observations on its efficacy in achieving natural, rejuvenated outcomes.

## METHODS

We conducted a retrospective observational study of all patients with dermatochalasis who underwent the MINEL procedure performed by a single surgeon (A.S.C.G.) from January 2021 to July 2021. The inclusion criteria encompassed patients with varying degrees of dermatochalasis who underwent the MINEL procedure, had at least a 6-month postoperative follow-up, and completed an online patient satisfaction questionnaire at 6 months. The questionnaire, developed by the authors, offered 3 response options: very satisfied, satisfied, and dissatisfied. Exclusion criteria encompassed patients with substantial redundant upper eyelid skin that could not be adequately lifted to ensure sufficient pretarsal platform exposure, as well as those who opted for skin excision to alleviate symptoms of heaviness.

In the MINEL procedure, precision is paramount, and each step is designed to minimize tissue damage while preserving the natural anatomy of the eyelid. The research was registered in the National Medical Research Register (NMRR ID-25-00965-M6F). This retrospective study, based on medical records or archived samples, received written informed consent from all patients. The study adhered to the guidelines set forth by the Declaration of Helsinki.

### Surgical Technique

**Figure 1.**
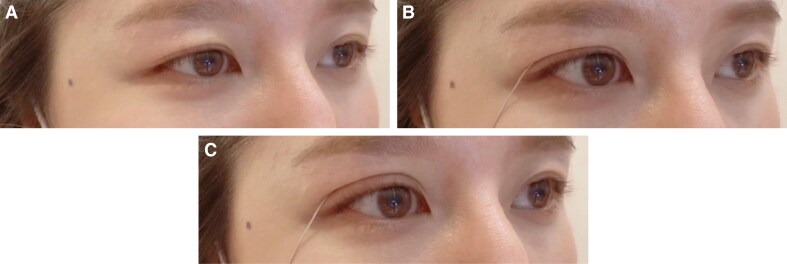
This sequence illustrates how the Minimally Invasive Eyelid Lift (MINEL) approach can be tailored to the patient's desired level of eyelid elevation without the need for skin excision. (A) A 40-year-old female presenting with mild dermatochalasis, characterized by redundant upper eyelid skin. (B) A Putterman eyelid creaser is used to gently tuck the excess skin inward, producing a mild elevation of the upper eyelid. (C) Additional skin is tucked inward, resulting in a more pronounced lift of the upper eyelid, showcasing the customizable nature of the MINEL technique.

**Figure 2.**
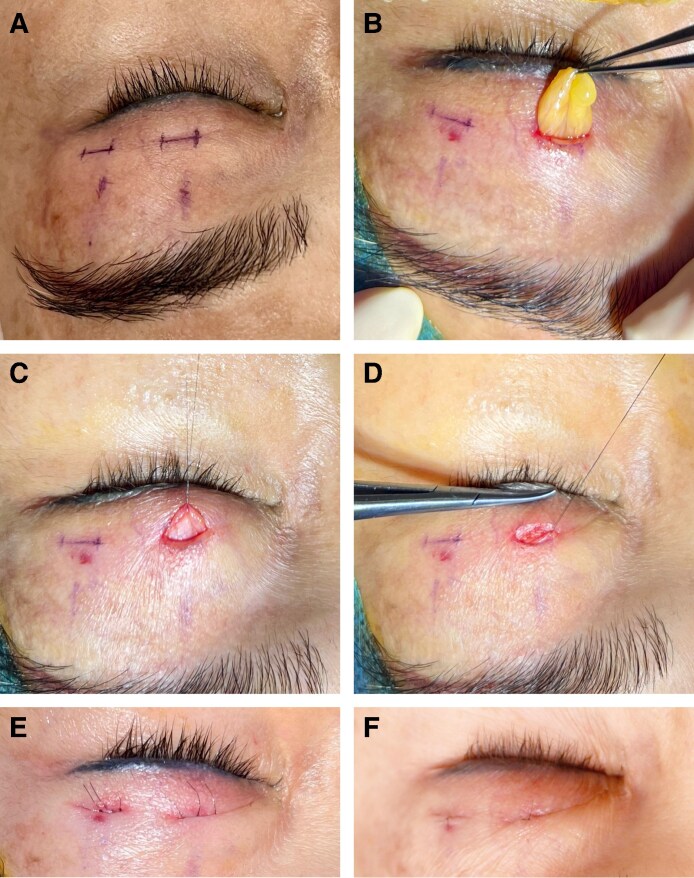
(A) Preoperative markings in a 60-year-old female patient indicating 2 small incisions (5 mm each) along the mid-pupillary line and an additional incision midway between the lateral limbus and the lateral canthus. These markings guide precise placement for minimal dissection. (B) Dissection exposes the preaponeurotic fat after clearing the pretarsal tissue and orbital septum, ensuring optimal visualization of the surgical plane. (C) A 7-0 nylon suture is carefully threaded through the levator aponeurosis to facilitate secure fixation and crease formation. (D) The suture is anchored to the pretarsal orbicularis muscle, creating a firm connection to enhance the eyelid contour and maintain long-term stability. (E) Three anchoring points are established within each incision, reinforcing the attachment between the levator aponeurosis and the pretarsal orbicularis muscle for uniform crease formation. (F) The incisions are meticulously closed using 7-0 nylon sutures, ensuring minimal scarring and a natural postoperative appearance.

Preoperative marking is performed with the patient in an upright position to ensure precise identification of the redundant skin fold. Putterman eyelid creaser is used to gently tuck the excess upper eyelid skin inward, facilitating accurate and consistent marking with a surgical marker. The amount of skin to be tucked is predetermined based on the desired tarsal platform show, tailored to each patient's anatomy and aesthetic goals (Figure 1). The first marking is placed at the mid-pupillary line, corresponding to the highest point of the new eyelid crease. A second mark is positioned midway between the lateral limbus and the lateral canthus (Figure 2A, Video).

MINEL procedures were performed under local anesthesia without sedation. A local anesthetic, typically lidocaine with epinephrine (0.2-0.5 cc Lidocaine 2% + 1:100,000 epinephrine per eyelid), is injected into the marked area to provide both anesthesia and vasoconstriction, reducing intraoperative bleeding. Careful infiltration is done to avoid tissue distortion during the procedure.

Using a No. 11 blade, small, precise incisions are made along the marked eyelid crease. These incisions are conservatively sized (typically 3-5 mm) to ensure minimal scarring while allowing sufficient access to the underlying tissue. Typically, 2 primary incisions are made in the upper eyelid crease to address hooding. However, in cases of significant skin laxity, additional incisions may be necessary to ensure a smooth and natural eyelid contour. The key to this technique lies in its tissue-sparing approach, with a focus on limiting unnecessary removal of skin and muscle.

Once the incision is made, Westcott scissors are used to carefully separate the orbicularis oculi muscle. This is done in a meticulous manner to avoid bleeding, and hemostasis is maintained throughout the procedure. The dissection is blunt, following the natural tissue planes, which reduces the risk of damage to surrounding structures. Hemostatic devices (Bovie, fine-tip high-temperature cautery from Bovie Medical and Bovie Property Group Ltd) are employed throughout the surgery to ensure a clean surgical field.

Through the small incisions, dissection proceeds layer by layer through the soft tissues until the orbital septum is exposed. Careful dissection along the septum ensures that the fat pads can be accessed without compromising other important structures such as the levator muscle. This precision is critical to avoid postoperative complications such as ptosis. Once the septum is reached, the underlying preaponeurotic fat is evaluated (Figure 2B). Depending on the patient's specific anatomical needs and aesthetic goals, the fat may either be preserved to maintain volume or selectively removed. In younger patients or those without significant fat herniation, preserving this fat can prevent the hollowed appearance often seen with traditional blepharoplasty. For patients with excess bulging fat or a prominent nasal fat pad, a conservative amount of fat is excised through an additional incision in the medial aspect to smoothen the eyelid contour while maintaining a natural look.

The key innovation of the MINEL technique is the use of a “tuck” rather than excision to reposition the excess skin. A fine suture, typically Nylon 7/0 (Mani, Lancet single-armed Nylon 7/0, PE11-33), is passed through a bite of the levator aponeurosis without dissecting it and secured to the orbicularis muscle (Figure 2C, D). Two to three anchoring points established in each incision between the levator aponeurosis and the pretarsal orbicularis muscle (Figure 2E). The levator aponeurosis is not dissected or released from tarsus. The level of anchoring of the levator aponeurosis depends on the height of the crease which can be up to 15 mm, because the levator aponeurosis extends somewhere around 14 to 20 mm from the superior tarsal border. This step effectively lifts the eyelid and tucks the redundant skin to a higher level, creating a more defined crease. The dynamic nature of the eyelid's movement is preserved, and the procedure mimics the natural function of the orbicularis–levator–aponeurosis complex. This tucking approach not only avoids visible scarring but also reduces the risks associated with skin excision, such as eyelid malposition or excessive hollowing. Once the sutures are placed and the skin is adequately tucked, the remaining skin is redraped over the newly formed crease. Because the excess skin is tucked rather than removed, this technique results in a natural, smooth appearance without tightness or over-pulling. Any small skin folds naturally settle over time, giving the eyelid a youthful, rejuvenated look.

The skin incisions are closed using fine, nonabsorbable sutures (Mani Lancet single-armed Nylon 7/0, PE33-11), which are removed after about 7 days postoperatively (Figure 2F). Suturing is done with meticulous care to ensure proper wound healing and to minimize the appearance of scars. Careful attention is given to the alignment of the skin edges to ensure optimal healing and cosmetic outcomes.

### Postoperative Care

Patients are advised to follow a gentle postsurgical care regimen, including antibiotic ointment to be applied to the surgical wound twice a day. Cold compresses are applied in the immediate postoperative period to reduce swelling and bruising. Most patients experience minimal discomfort, and oral analgesics are prescribed as needed. Patients can resume their normal activities relatively quickly, with most reporting a return to their usual routine within 1 to 2 weeks. Makeup can usually be applied after about 7 days to cover any residual bruising or swelling.

## RESULTS

In a preliminary assessment of 37 patients who underwent the MINEL procedure. The study population included both male (*n* = 6) and female patients (*n* = 31) aged 40 to 71 years (mean age: 47), presenting with different levels of upper eyelid aging. The cohort consists of 34 Malaysian, 1 Singaporean, 1 Indonesian, and 1 Canadian (Table 1).

**Table 1.** Demographics and Age Distributions

	Count	Percentage
Age group, years		
40-49	13	35.1
50-59	16	43.2
60+	8	21.6
Gender		
Female	31	83.8
Male	6	16.2
Nationality		
Malaysian	34	91.9
Singaporen	1	2.7
Indonesian	1	2.7
Canadian	1	2.7

**Figure 3.**
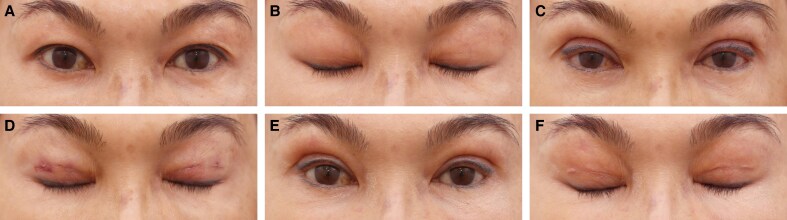
(A, B) Preoperative images of a 60-year-old Malaysian woman presenting for upper blepharoplasty, with the image showing the downward gaze to assess upper eyelid hooding. (C, D) Immediate postoperative results following the Minimally Invasive Eyelid Lift technique, demonstrating skin repositioning with minimal trauma. The corresponding downward gaze image highlights the initial wound closure. (E, F) One-week postprocedure, showing progressive healing with well-approximated incision lines. The image in the downward gaze further confirms early recovery with mild residual swelling.

**Figure 4.**
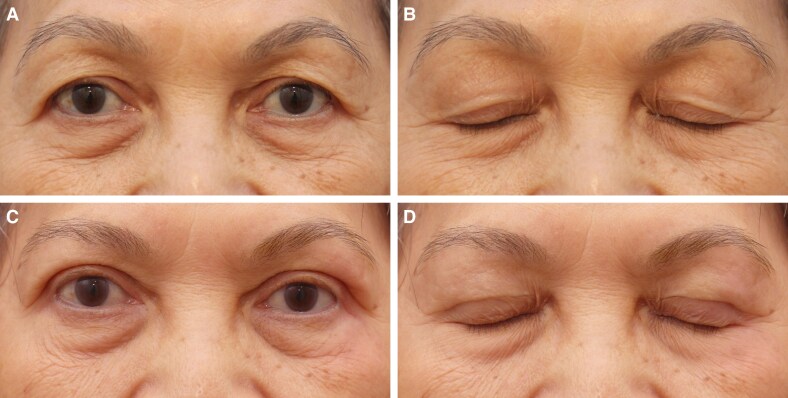
(A, B) Preoperative images of a 65-year-old Malaysian woman with sunken upper sulci and dermatochalasis seeking upper blepharoplasty. The corresponding downward gaze image further illustrates the volume deficiency and skin laxity. (C, D) Eight months postoperatively following the Minimally Invasive Eyelid Lift technique, demonstrating significant improvement in upper eyelid contour with restoration of volume and reduction of dermatochalasis. The corresponding downward gaze image confirms natural crease formation and a smooth upper eyelid transition.

**Figure 5.**
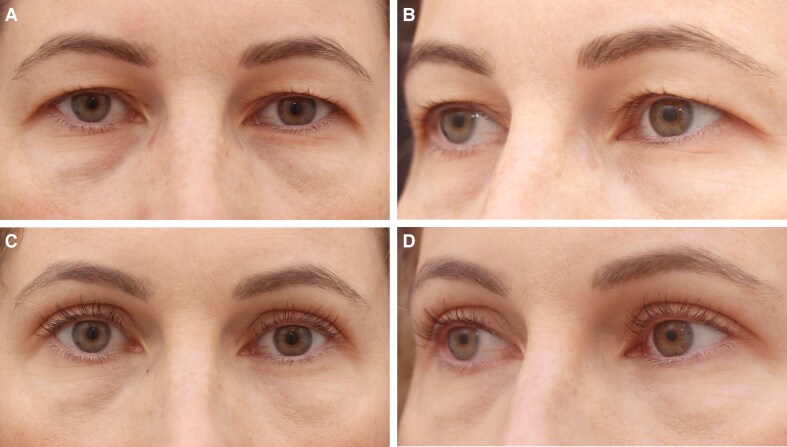
(A, B) Preoperative images of a 44-year-old Canadian woman seeking upper blepharoplasty, highlighting preexisting eyelid skin redundancy and sulcus hollowing. (C, D) One-month postoperative images following the Minimally Invasive Eyelid Lift technique highlight its effectiveness in Occidental patients, showing enhanced upper eyelid contour and restored volume in the superior sulcus, with minimal signs of trauma during the early recovery phase.

**Figure 6.**
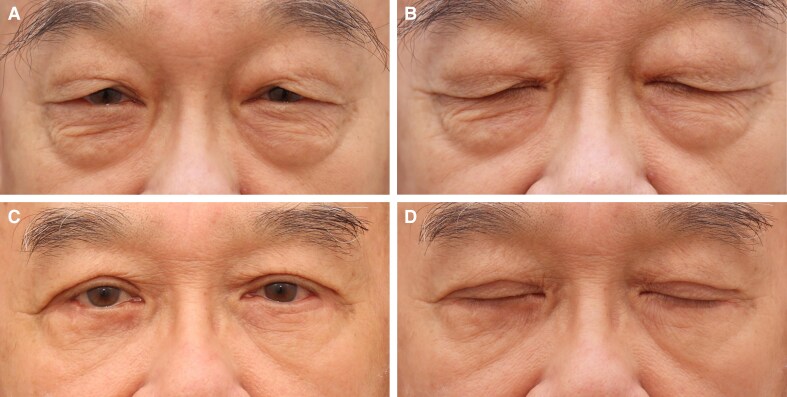
(A, B) Preoperative images of a 72-year-old Malaysian gentleman with significant dermatochalasis, leading to visual field obstruction and an aged appearance. The images illustrate the patient's eyelid position with eyes closed. (C, D) One-year postoperative images following the Minimally Invasive Eyelid Lift technique, demonstrating a well-defined upper eyelid crease, improved eye-opening, and a rejuvenated periorbital appearance. The corresponding eye-closed view highlights the preservation of natural eyelid dynamics with minimal signs of surgical intervention, confirming the long-term efficacy and stability of the results.

All patients (37/37, 100%) reported satisfaction with the overall functional and aesthetic outcomes of the MINEL procedure. Of these, 70% were very satisfied, and 30% were satisfied, with the eyelids achieving a naturally rejuvenated appearance and a smoother, more youthful contour. The tuck technique effectively addressed redundant skin while enhancing periorbital harmony (as demonstrated in Figures 3-6). Notably, 92% of the patients (34/37) reported that their natural eyelid crease was preserved, with either no visible scarring or scars that were barely noticeable.

The average MINEL surgery time was 33.9 min, with 48.6% of procedures completed in ≤30 min and 51.4% in 31 to 60 min. Recovery times were notably quicker, with 90% of patients returning to normal activities within 7 days. The overall recovery period ranged from 3 to 10 days.

The most frequently observed complications after the MINEL procedure included crease asymmetry requiring postoperative adjustment in 5 patients (13.5%) and upper eyelid bulkiness in 1 patient (2.7%), who subsequently underwent full-incision upper blepharoplasty.

## DISCUSSION

We emphasize the growing importance of tissue preservation in aesthetic surgery, particularly in upper blepharoplasty, where the goal is to achieve natural and long-lasting results. Traditional excision-based techniques, while effective, may lead to complications such as eyelid malposition, hollowing, and visible scarring, especially in patients with minimal skin redundancy.^1,4,9^ In contrast, the MINEL procedure focuses on preserving tissue by tucking excess skin inward, which reduces surgical trauma and allows for dynamic crease formation that mimics natural eyelid folds (Figures 3-6, Supplemental Figures 1, 2). Ideal candidates are those seeking enhancement of their upper eyelid appearance without the risks associated with more extensive surgery.

MINEL's technical simplicity and reproducibility are highlighted, making it an accessible option for surgeons familiar with upper blepharoplasty. By avoiding extensive dissection, MINEL minimizes tissue injury and provides more control over intraoperative adjustments, offering a tailored outcome for each patient. This makes it a preferable choice when compared with traditional approaches, where determining the correct amount of skin to excise can be challenging, especially in patients with dermatochalasis (Figures 3-6).

The technique's emphasis on preserving the natural aesthetic of the upper eyelid is another key benefit, because it avoids the “hollowed” appearance that can result from more aggressive procedures. By maintaining eyelid fullness and structure, MINEL produces more subtle, harmonious results that align with patients’ preferences for a natural enhancement. Furthermore, preserving fat and orbicularis oculi is important for achieving youthful fullness and preventing issues such as a sunken sulcus, which can occur with aging or prior surgeries. The preservation of skin and soft tissue can help prevent exacerbating this condition and, in some cases, can improve the existing sulcus (Figure 4).

Although some surgeons may perceive the postoperative lid crease as being aesthetically high, the MINEL technique does not prioritize final crease height as the main indicator of success. Instead, the ideal aesthetic result is determined by the desired tarsal platform show—typically between 3 and 5 mm—tailored to the patient's personal preference. To achieve this, both the intended platform and the amount of skin to be tucked are carefully evaluated and planned during the preoperative stage. MINEL's innovation lies in its ability to restore and reinforce the eyelid crease, particularly in patients with age-related involutional changes. These changes can weaken the natural crease, leading to a flattened or less defined appearance. In such cases, MINEL can be applied to enhance and stabilize the existing crease.

Unlike traditional techniques that focus on skin excision, MINEL addresses underlying connective tissue and muscles to rejuvenate the eyelid's appearance without removing excess skin. This provides a more refined and youthful contour, even in patients with preexisting eyelid creases. The technique effectively addresses age-related changes that result in a flattened or less defined eyelid crease (Figure 5).

Additionally, MINEL is suitable for patients of all races, ages, and genders, offering a versatile approach to treating upper eyelid redundancy. It is effective in creating a natural, rejuvenated look for patients with varying anatomical features. The procedure can be successfully performed in Occidental patients, tucking up to 15 mm of skin while achieving a rejuvenated and natural appearance (Figure 5).

However, common complications of the MINEL procedure include crease asymmetry, upper eyelid bulkiness, and an unintended crease height, either higher or lower than expected. In such cases, touch-up surgery may be performed 4 to 6 months after the initial procedure to refine the results. A small number of patients developed intraoperative iatrogenic ptosis, likely because of inadvertent injury to the levator aponeurosis caused by over-dissection or taking an excessively large bite of the aponeurosis when anchoring to the orbicularis oculi muscle, potentially impairing its function. However, in all instances, this was promptly corrected during surgery by releasing the previous suture and re-stitching, effectively restoring normal eyelid function and contour and preventing any postoperative ptosis.

From both the surgeon's and patient's perspectives, MINEL offers significant advantages. The procedure is less invasive, reducing operating time, postoperative complications, and recovery time. Surgeons can achieve better results with fewer risks, while patients benefit from the quick recovery, minimal scarring, and subtle, natural enhancements. The dynamic nature of the crease formation and the ease of intraoperative adjustments give surgeons greater control over the final result. These factors collectively reduce the cost of the procedure and improve patient outcomes by limiting downtime and discomfort. Moreover, the procedure's minimally invasive nature results in higher patient satisfaction. In our study, 100% of patients reported being satisfied with the MINEL procedure, as patients appreciate the subtle improvements without looking as though they have undergone extensive surgery. Additionally, the absence of large incisions or visible scars contributes to a higher level of satisfaction, particularly among those seeking a conservative enhancement (Figures 3-6).

Overall, MINEL presents a promising alternative to traditional upper blepharoplasty techniques, prioritizing tissue preservation and offering high patient satisfaction, with reduced risk of complications and a more natural, youthful appearance.

### Comparative Studies and the Future of Blepharoplasty

The principles of tissue preservation and minimally invasive surgery are gaining widespread acceptance in facial cosmetic surgery, particularly in blepharoplasty. Studies on other minimally invasive techniques, such as fat grafting in facial rejuvenation, have demonstrated the importance of volume preservation in achieving natural, youthful results. Hamra's 1992 findings on composite facelift and extended blepharoplasty techniques highlight the benefits of tissue preservation and repositioning, as opposed to excision, in achieving more balanced and harmonious facial contours.^10^ Such evidence further supports the tissue-conservation approach adopted by the MINEL technique.

Further advancements in nonsurgical and minimally invasive eyelid rejuvenation techniques may also influence the future of blepharoplasty. Injectable fillers, skin-tightening devices, and energy-based treatments, such as radiofrequency and ultrasound, are being increasingly utilized for mild eyelid laxity, offering alternatives to surgery altogether. However, for patients with moderate-to-significant skin excess, surgical approaches like MINEL may remain the best option. With time, the role of MINEL in the broader landscape of upper blepharoplasty will become clearer as more research and case studies emerge. 

### Limitations and Patient Selection Considerations

Limitations of this study include the small cohort size, its single-surgeon and retrospective nature, and its lack of objective and validated outcome measures. The MINEL procedure itself has certain limitations. First, visualizing deeper eyelid structures through small incisions can be challenging, increasing the risk of inadvertent injury to the levator aponeurosis, which may result in iatrogenic ptosis. Additionally, in patients with naturally bulky eyelids, the skin-tucking technique can create an appearance of increased bulkiness, which may not be aesthetically acceptable to some individuals. Finally, this approach is not suitable for patients with significant excess skin, as the technique may not effectively address their concerns.

## CONCLUSIONS

The MINEL technique represents a promising alternative to traditional excision-based upper blepharoplasty. To the best of our knowledge, this is the first paper that describes a skin-preservation upper blepharoplasty. By prioritizing tissue preservation and minimizing invasiveness, MINEL provides patients with natural-looking results, minimal scarring, and shorter recovery times. This approach offers particular advantages in patients seeking subtle aesthetic enhancement without the overt signs of surgery. Because trends in cosmetic surgery continue to evolve, the tissue-preservation philosophy exemplified by MINEL is likely to gain further prominence.

## Supplementary Material

ojaf107_Supplementary_Data
