# Supplementary figures and images for: Minimally Invasive Eyelid Lift: A Skin Preservation Upper Blepharoplasty
Source: Aesthet Surg J Open Forum. 2025 Aug 25;7:ojaf107. doi: 10.1093/asjof/ojaf107 (PMC12548048; doi:10.1093/asjof/ojaf107)

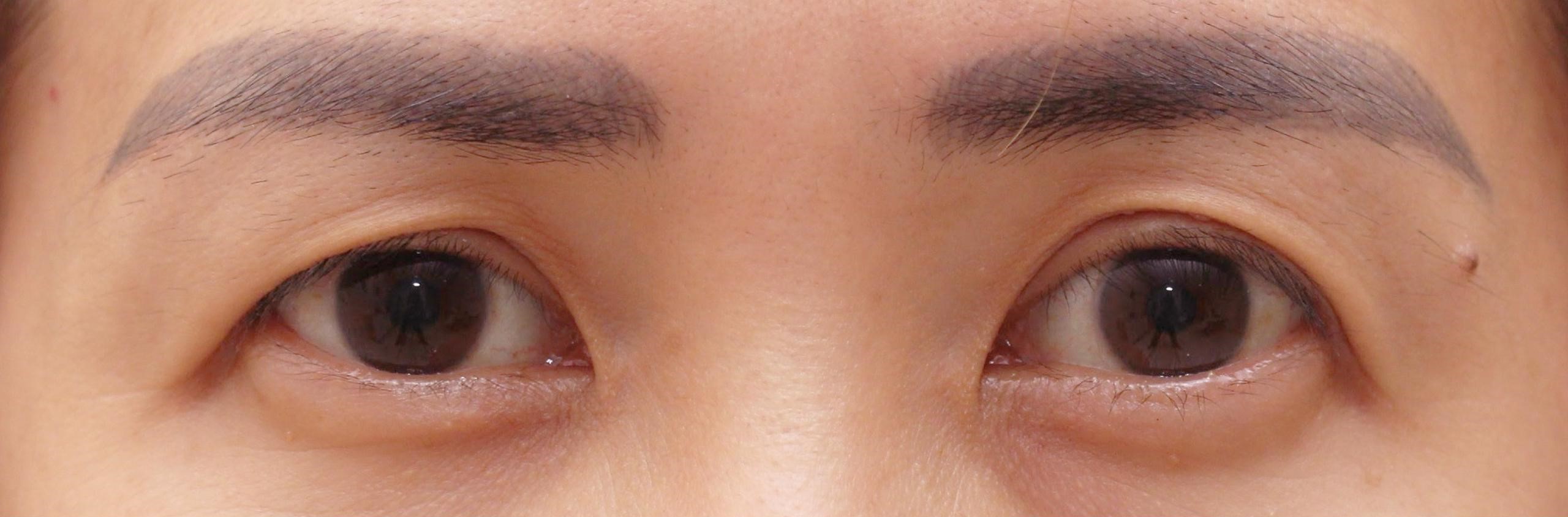

Supplement: ojaf107_Supplementary_Data [file ojaf107_Supplementary_Data.zip › Supplementary Figure 1 A.jpg]

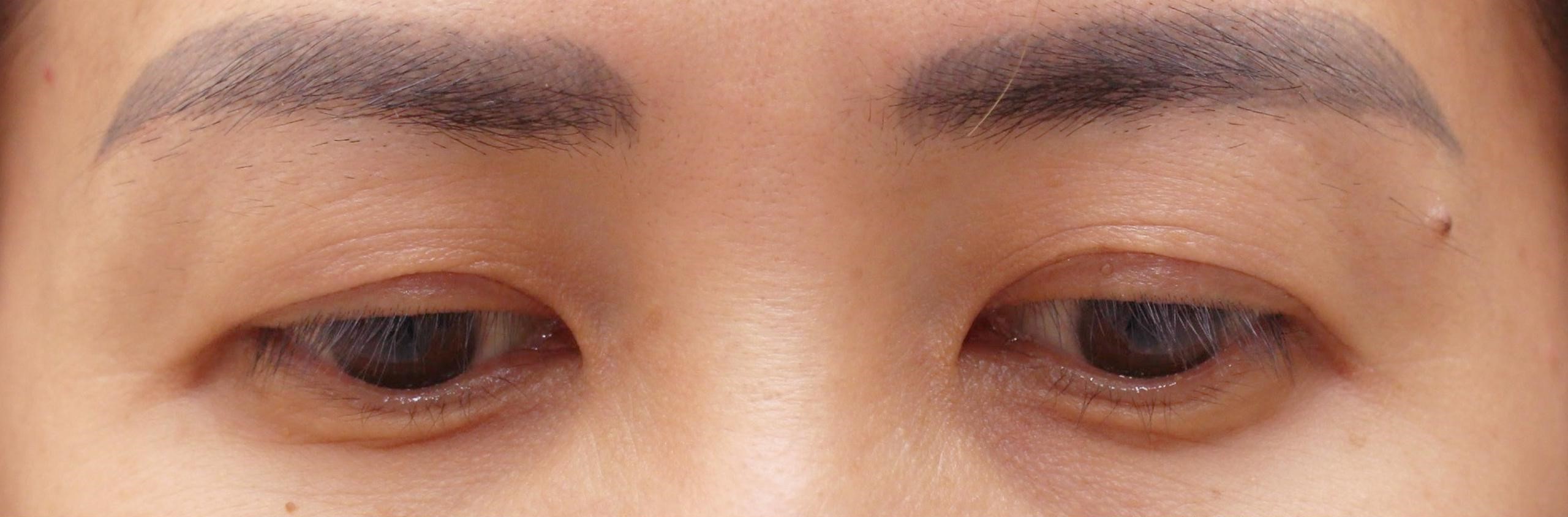

Supplement: ojaf107_Supplementary_Data [file ojaf107_Supplementary_Data.zip › Supplementary Figure 1 B.jpg]

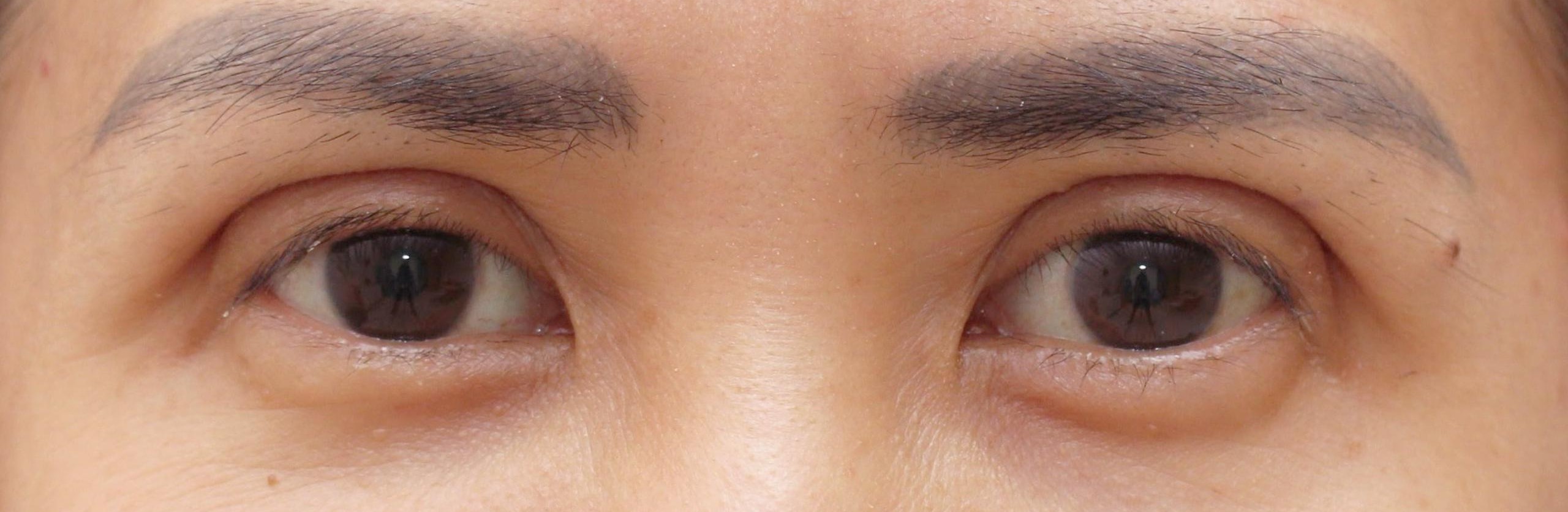

Supplement: ojaf107_Supplementary_Data [file ojaf107_Supplementary_Data.zip › Supplementary Figure 1 C.jpg]

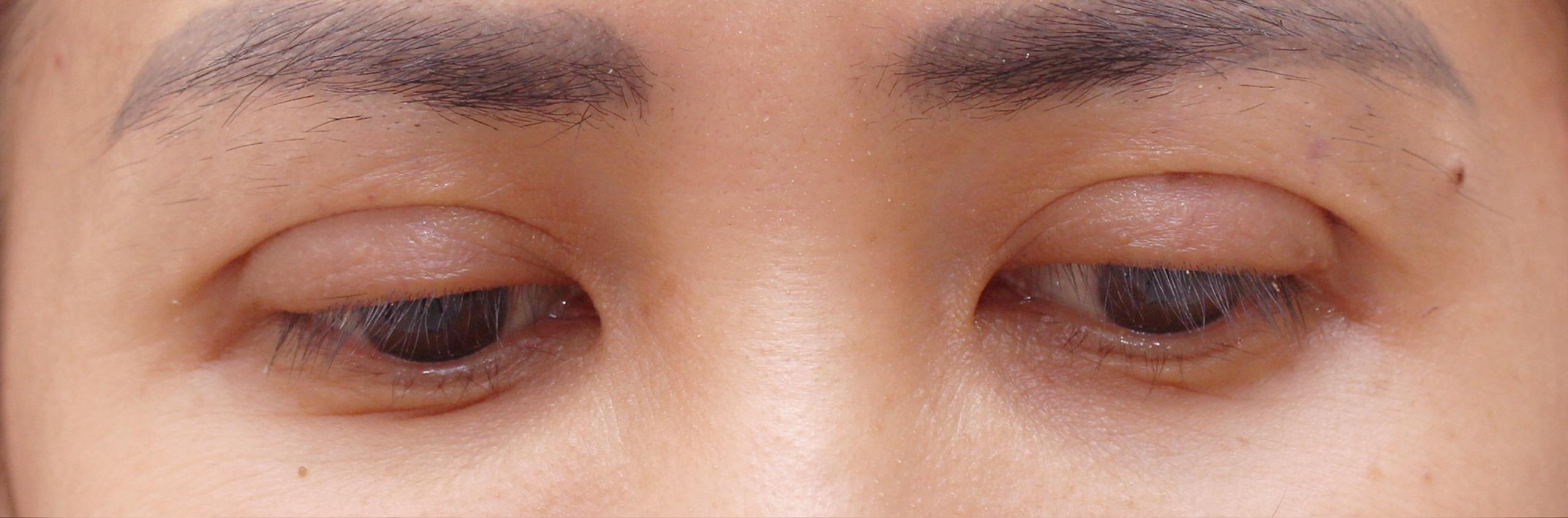

Supplement: ojaf107_Supplementary_Data [file ojaf107_Supplementary_Data.zip › Supplementary Figure 1 D.jpg]

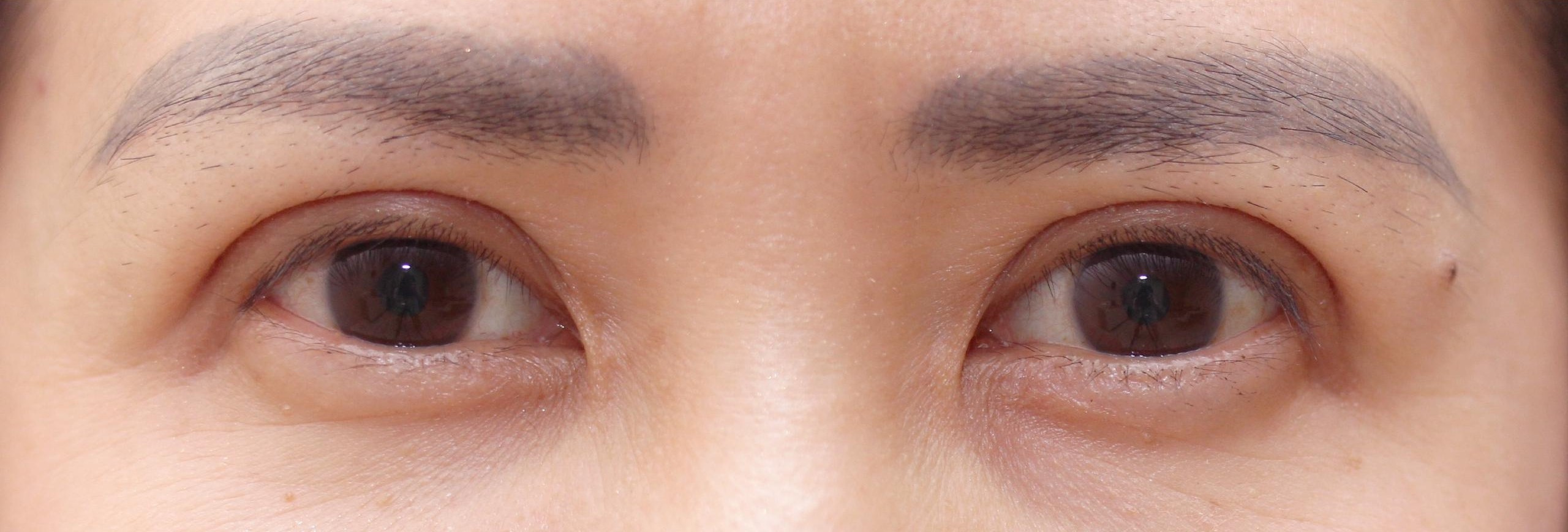

Supplement: ojaf107_Supplementary_Data [file ojaf107_Supplementary_Data.zip › Supplementary Figure 1 E.jpg]

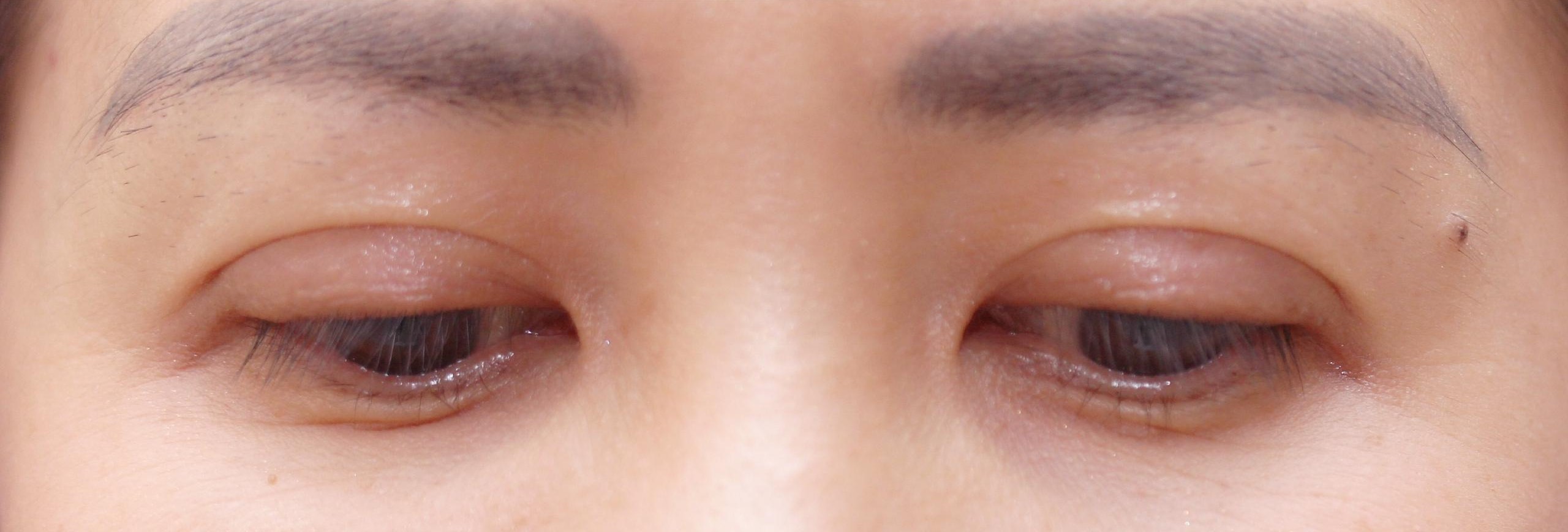

Supplement: ojaf107_Supplementary_Data [file ojaf107_Supplementary_Data.zip › Supplementary Figure 1 F.jpg]

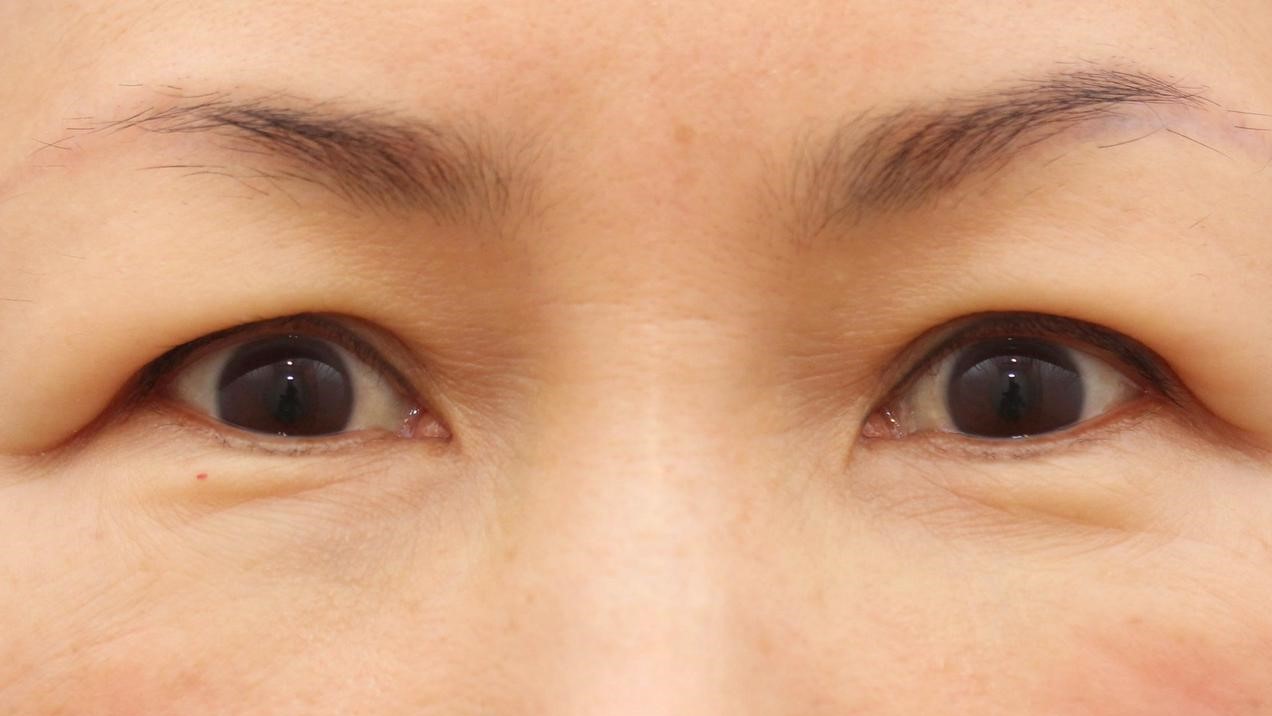

Supplement: ojaf107_Supplementary_Data [file ojaf107_Supplementary_Data.zip › Supplementary Figure 2 A.jpg]

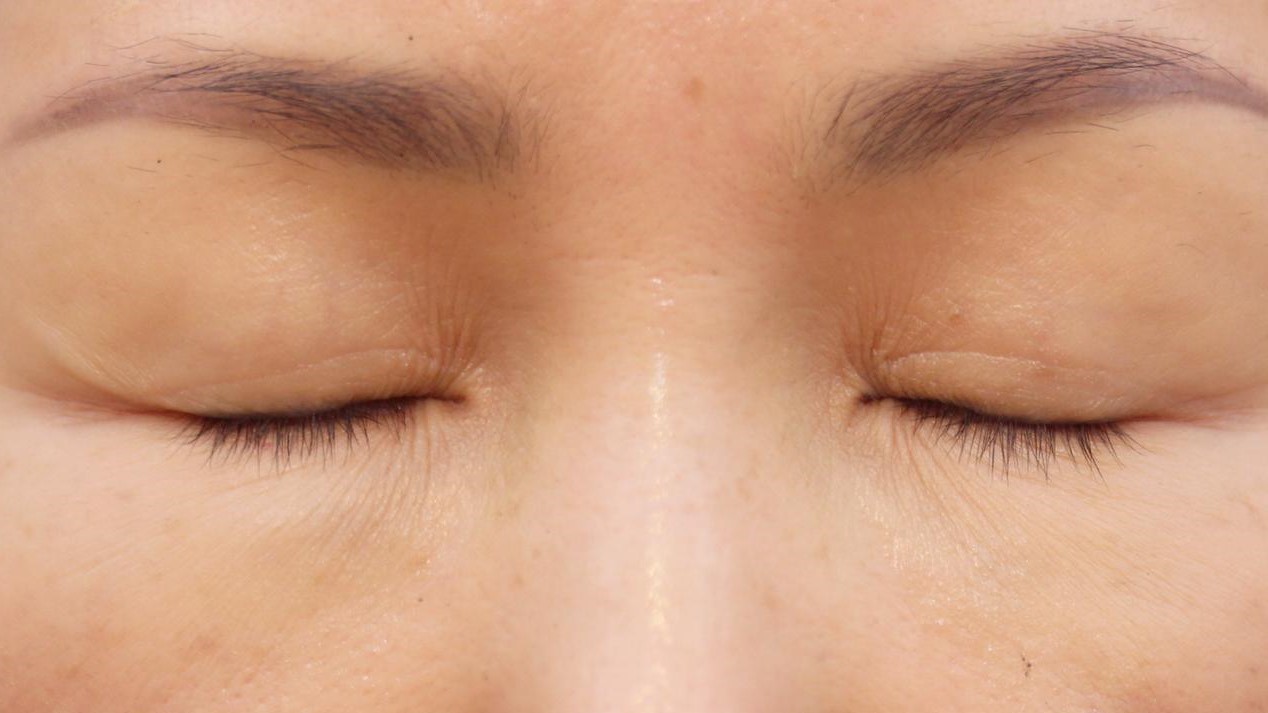

Supplement: ojaf107_Supplementary_Data [file ojaf107_Supplementary_Data.zip › Supplementary Figure 2 B.jpg]

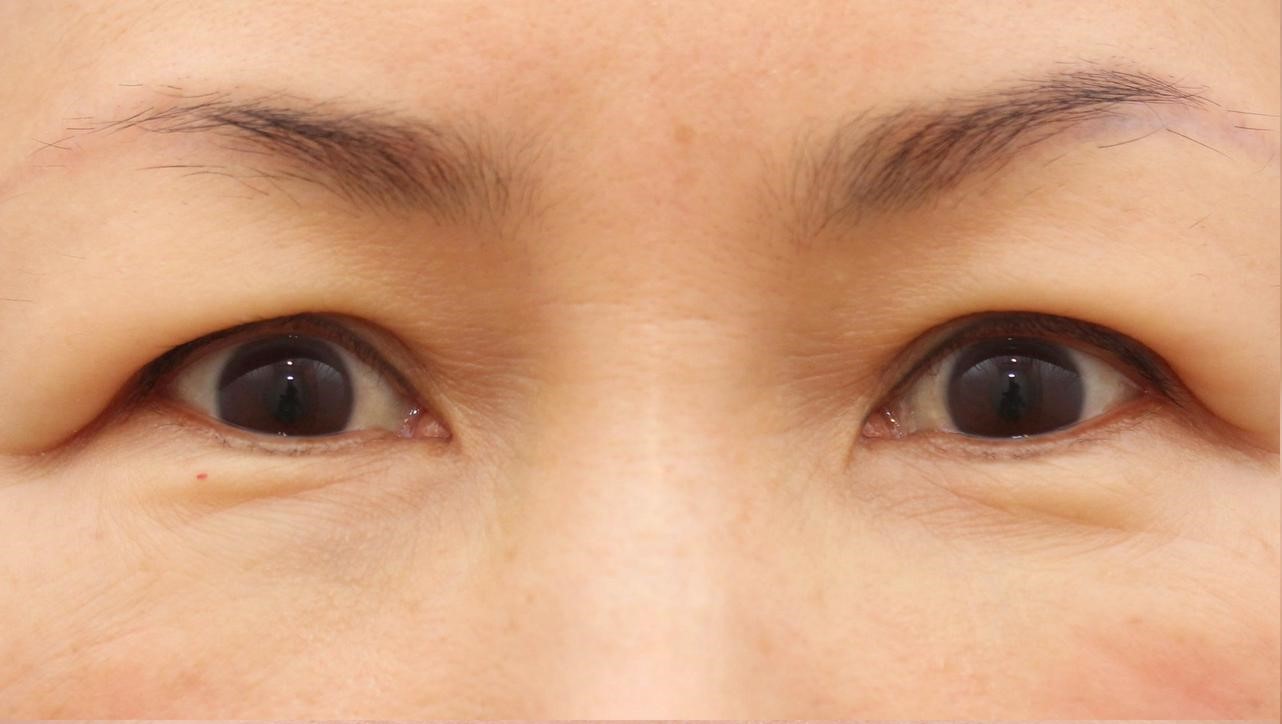

Supplement: ojaf107_Supplementary_Data [file ojaf107_Supplementary_Data.zip › Supplementary Figure 2 C.jpg]

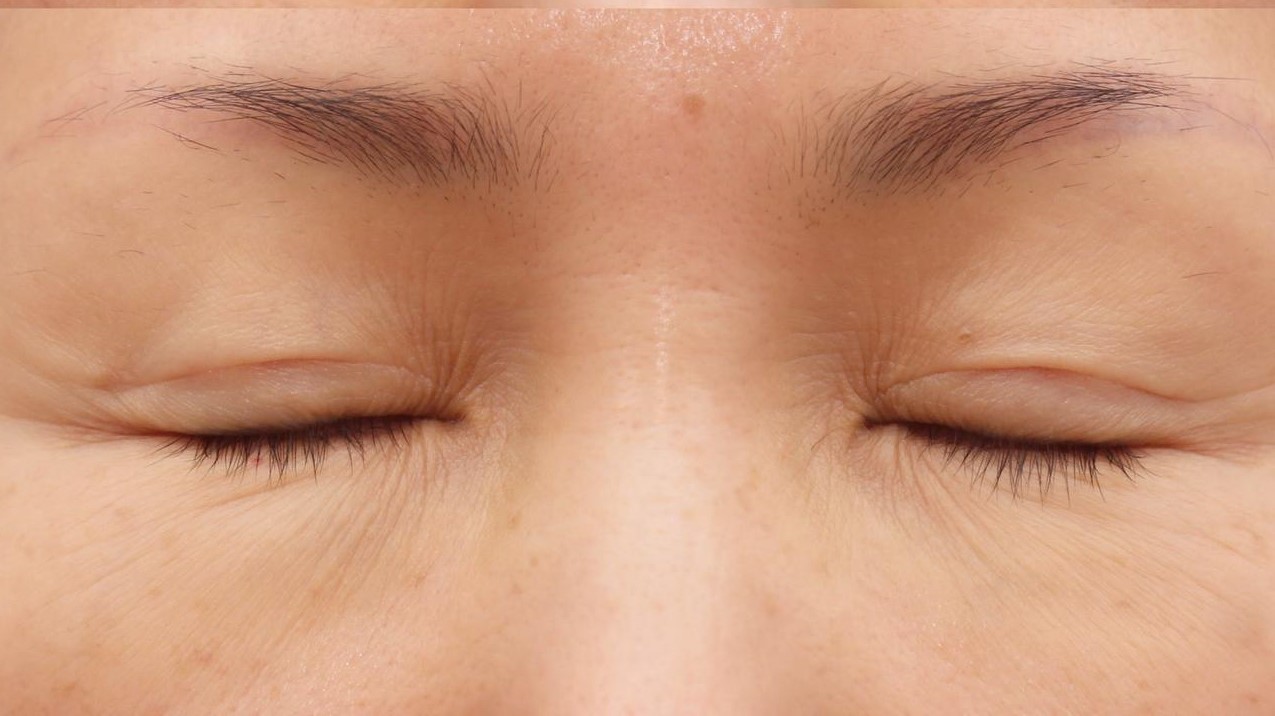

Supplement: ojaf107_Supplementary_Data [file ojaf107_Supplementary_Data.zip › Supplementary Figure 2 D.jpg]
